# Distinct changes in the colonic microbiome associated with acute diverticulitis

**DOI:** 10.1111/codi.16271

**Published:** 2022-08-11

**Authors:** O’Grady MJ, Greg A. Turner, Sulit A, Frank A. Frizelle, Purcell R

**Affiliations:** ^1^ Whanganui Hospital Wanganui New Zealand; ^2^ Royal Melbourne Hospital Parkville Victoria Australia; ^3^ University of Otago Christchurch New Zealand; ^4^ Christchurch Hospital Christchurch New Zealand

**Keywords:** acute diverticulitis, colonic Mircobiome treatment, microbiom

## Abstract

**Aim:**

The pathogenesis of acute diverticulitis (AD) remains incompletely understood, despite it being one of the most common gastrointestinal conditions worldwide. The aim of this study was to investigate the role of the colonic microbiome in the pathogenesis of AD.

**Method:**

A prospective case–control study was performed, comparing the microbiome of AD patients with that of controls, using 16S rRNA sequencing of rectal swab samples.

**Results:**

The microbiome of individuals with AD showed lower diversity than that of controls. There were significant compositional differences observed, with a lower abundance of commensal bacterial families and genera such as *Lachnospiraceae*, *Ruminococcus* and *Faecalibacterium* in AD patients compared with controls*,* and there was an increase in several genera with known pathogenic roles including *Fusobacteria*, *Prevotella* and *Paraprevotella*.

**Conclusion:**

This is the largest study to date to examine the microbiota of AD patients, and adds evidence to the proposed hypothesis that alterations in the colonic microbiome play a role in the pathogenesis of AD.

**What does this paper add to the literature?**

This paper adds evidence for the proposal hypothesizing that alterations in the colonic microbiome play a role in the pathogenesis of acute diverticulitis (AD). While this is a challenging area to study, improved understanding of the potential role of the microbiota in the pathogenesis of AD has significant clinical importance and warrants further investigation.

## INTRODUCTION

Acute diverticulitis (AD) is a disease with a significant impact on global health and resources [1]. This condition is characterized by peri‐colonic inflammation arising in an outpouching (diverticulum) in the colonic wall. Complicated AD, defined by the modified Hinchey classification (Stages Ib–IV) [2] as the presence of perforation, abscess, fistula or purulent or faeculent peritonitis, can lead to systemic sepsis and the requirement for operative intervention [3]. Aging populations, global uptake of a Western‐style diet and lifestyles, and increasing incidence in younger people are driving a surge in incidence [4]. The requisite precursor for AD, diverticulosis, is an asymptomatic condition with a lifetime prevalence of up to 72% [5]. However, fewer than 5% of patients with diverticulosis go on to develop AD, and the reasons why this small proportion progress are unclear [6]. Recent research has indicated a genetic contribution, while environmental risk factors, including diet, sedentary lifestyle, obesity, smoking and medications, have been repeatedly demonstrated in large correlational studies [7, 8, 9, 10, 11, 12]. The exact pathogenic mechanism through which these risk factors exert their effect is again unknown, but current theory suggests that these factors impact the gut microbiota and its interplay with the human host [13].

Research findings over the last 10 years have demonstrated the importance of the gut microbiota and its impact on human health. The gut microbiota influences colonic mucosal defences and local and systemic inflammation. A role in the pathogenesis of gastrointestinal diseases, including *Clostridium difficile* colitis, inflammatory bowel disease and irritable bowel syndrome, has been confirmed, while several small studies have indicated a role of the microbiota in AD [14]. Dysbiosis of the microbiome, either locally within a diverticulum or throughout the colon, could lower the threshold for an initiating stimulus, such as impaction of a faecolith, to trigger the pathway to AD.

The aim of this study is to compare the gut microbiota of individuals with AD with that of controls. Demonstrating a link between AD and the gut microbiota would advance our understanding of this disease and has the potential to provide an opportunity for prevention of both primary and recurrent disease and aid clinical decision‐making with regard to elective colonic resection to prevent future attacks.

## METHOD

### Study design

A prospective, single‐centre case–control study was performed comparing the microbiome of patients with AD (Hinchey Ia–IV) with that of controls without signs of diverticulitis. All adult (>18 years) patients admitted to Christchurch Hospital, New Zealand between 1 February and 31 August 2020 with a diagnosis of AD were assessed for eligibility for the study. Diagnosis of AD was confirmed by CT scan. The decision to admit to hospital was at the discretion of the clinical team but generally followed existing literature and guidelines. Patients with uncomplicated disease (Hinchey 1a), absence of comorbidities or immunocompromised state, with the ability to tolerate oral intake and an adequate social situation, were considered for outpatient management. All others were admitted and were eligible for inclusion. Exclusion criteria were those patients who had antibiotics in the 6 weeks preceding admission (excepting antibiotics commenced at the current admission, within 24 h of obtaining the rectal swab), mechanical bowel preparation (MBP) in the preceding 6 weeks, known or subsequent diagnosis of colorectal cancer or inflammatory bowel disease or patients unable to consent. Subgroup analysis of uncomplicated (Hinchey Ia) and complicated AD (Hinchey Ib–IV) was done.

Controls were adults without a previous history of AD. This cohort included people from a previous feasibility study run by our group and also individuals who were admitted to hospital with a diagnosis that was unrelated to the gastrointestinal system, for example those admitted for hernia repair or trauma‐related injury.

Rectal samples were taken from cases and controls using DNA/RNA Shield Collection tubes with swabs (Zymo Research). These swabs were placed in a DNA/RNA Shield Collection tube, prefilled with a solution that preserves DNA and RNA at ambient temperature.

### DNA isolation and amplification

DNA for microbiome analysis was extracted from the swabs using a ZymoBIOMICS DNA Kit (Zymo Research) according to the manufacturer’s instructions. DNA concentration and purity were assessed using a NanoDrop 2000 spectrophotometer (Thermo Fisher Scientific).

### 16S rRNA gene sequencing

Microbiome analysis was carried out using 16S rRNA sequencing. A Nextera® XT DNA Index kit (Illumina) was used for library preparation using a single‐step polymerase chain reaction (PCR) library preparation method that has dual‐index PCR primers that flank the V3–V4 hyper‐variable region of the 16S rRNA gene (16SF_V3: 5′‐TATG GTAATTGGCCTACGGGAGGCAGCAG‐3′ and 16SR_V4: 5′‐AGTCAGTCAGCCGGACTACHVGGGTWTCTAAT‐3′), and Illumina sequencing adaptors and barcodes were added using limited‐cycle PCR. The libraries were pooled by equal molarity before loading on the Illumina MiSeq platform with PhiX as 20% of the library and paired‐end reads of length 250 bp were generated.

### Bioinformatics analyses

#### Quality control

DADA2 (v.1.18.0) was used to filter, trim, join sequencing reads and remove chimeras to obtain amplicon sequence variants [15]. The parameters for filtering and trimming are shown in Table S1. The SILVA database (v.132) was utilized to assign taxonomy to the amplicon sequence variants, with the tryRC option set to True and utilizing the silva_sp and addSpecies commands to identify species‐level taxonomy, where possible [16]. The taxonomy, sample metadata and sequences were combined into a phyloseq object for subsequent analysis [17]. The associated code is available at https://gitlab.com/alsulit08/uoc_diverticulitis.

#### Diversity analyses

For alpha diversity, we rarefied all samples to the smallest library size of 11,659 reads. We used phyloseq’s estimate richness function for Observed (richness) and Shannon (richness and evenness) measures, and compared differences in the alpha diversity measures by Wilcoxon tests. A *p*‐value <0.05 was considered significant. We also used principal component analysis to test for overall differences in the microbiome between groups. We normalized our samples using centred‐log ratios through the microbiome R package, and performed ordination using phyloseq’s ordinate function with the ‘RDA’ option [18]. Significance was then calculated through Permanova using the adonis() function of the vegan package [19], and betadisper() to compare dispersions between groups.

#### Differential abundance

We used DESEq2 to obtain differentially abundant genera between the following comparisons: (1) AD versus control, (2) complicated AD versus uncomplicated AD, (3) complicated AD versus control and (4) uncomplicated AD versus control [20]. Sequencing of samples was performed in two batches, and we therefore used batch as a covariate in our design formula. We considered a fold‐change between comparisons to be significant if it has a *p*‐adjust value <0.05.

Detailed analysis of the sequences may be accessed at https://gitlab.com/alsulit08/ uoc_diverticulitis.

### Metadata analysis

To evaluate differences in baseline patient characteristics, Fisher’s exact and chi‐square tests were used for categorical data, with the Mann–Whitney *U*‐test and *t*‐test used for continuous data where appropriate. A *p*‐value of <0.05 was considered statistically significant.

## RESULTS

### Patient characteristics

During the study period, 120 patients were admitted with confirmed AD. A total of 65 patients with AD were included, 21 of whom had complicated disease. The reasons for exclusion were antibiotic use, either in the preceding 6 weeks (*n* = 16) or for more than 24 h for the current illness (*n* = 18), the patient declining to participate (*n* = 14), the patient was unable to consent (*n* = 2), immunosuppression (*n* = 2) and recent bowel preparation (*n* = 1). One individual with complicated AD agreed to participate but was excluded from analysis as insufficient DNA was extracted from the rectal swab. A total of 27 controls were included. The baseline demographics for each group are shown in Table 1 and for subgroups of uncomplicated and complicated AD in Table 2.

**TABLE 1:** Baseline patient characteristics: acute diverticulitis compared with controls

	Acute diverticulitis (*n* = 65)	Control group (*n* = 27)	*p‐*value
Female (%)	27 (42)	13 (48)	0.28
Age (years) (range)	58 (27–91)	46 (26–88)	<0.01
Smoker (%)	10 (15)	2 (7)	0.25

**TABLE 2:** Baseline patient characteristics of the acute diverticulitis group compared by disease classification

	Uncomplicated AD (*n* = 44)	Complicated AD (*n* = 21)	*p*‐value
Age (years) (range)	56 (27–91)	57 (39–78)	0.52
Female (%)	23 (52.3%)	5 (23.8%)	0.04
Mean BMI (kg/m^2^) (SD)	30.7 (5.0)	29.0 (4.0)	0.50
Comorbidity			
ASA grade			
1	17 (38.6%)	7 (33.3%)	0.56
2	22 (50.0%)	13 (61.9%)	
3	5 (11.4%)	1 (4.8%)	
4	0 (0%)	0 (0%)	
Modified Hinchey classification			
Ia	44 (100%)	0 (0%)	N/A
Ib	0 (0%)	19 (90.4%)	
II	0 (0%)	1 (4.8%)	
III	0 (0%)	1 (4.8%)	
IV	0 (0%)	0 (0%)	
Current smoker	6 (13.6%)	4 (19.0%)	0.72
Ischaemic heart disease	6 (13.6%)	1 (4.8%)	0.41
Pulmonary disease	5 (11.4%)	3 (14.3%)	0.70
Renal impairment	1 (2.3%)	0 (0%)	1.00
Diabetes	4 (9.1%)	1 (4.8%)	1.00
Immunosuppression	1 (2.3%)	3 (14.3%)	0.10
Previous colonic resection	1 (2.3%)	0 (0%)	1.00
Previous appendicectomy	6 (13.6%)	5 (23.8%)	0.31
Mean CRP at admission (SD)	72.5 (49.5)	132.5 (83.2)	<0.01
Mean WCC at admission (SD)	12.2 (3.2)	13.7 (3.1)	0.09
Duration of symptoms (days) (range)	2 (1–14)	2 (1–40)	0.49
Previous episode of AD	9 (20.5%)	8 (38.1%)	0.15

Abbreviations: AD, acute diverticulitis; ASA, American Society of Anesthesiologists; BMI, body mass index; CRP, serum C‐reactive protein (mg/L); WCC, white blood cell count (×10^9^/L).

### Alpha diversity

**FIGURE 1:**
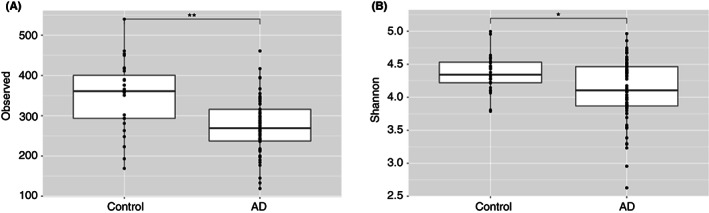
Observed (A) and Shannon (B) alpha diversities between acute diverticulitis and controls. *p*‐value: *<0.05; **≤0.01; ***≤0.001.

We compared Observed and Shannon measures of alpha diversity between control and AD samples. There was a statistically significant decrease in Observed alpha diversity and Shannon diversity index, *p* = <0.001 and *p* = 0.019, respectively, in AD compared with controls (Figure 1).

**FIGURE 2:**
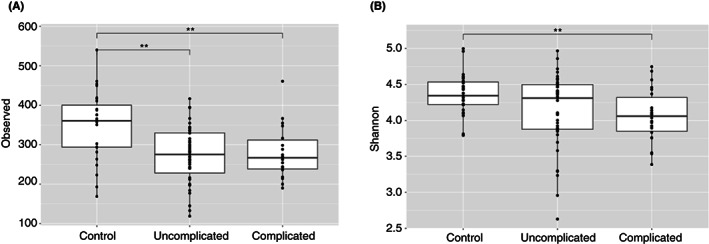
Observed (A) and Shannon (B) alpha diversities between uncomplicated, complicated and control groups. *p*‐value: *<0.05; **≤0.01; ***≤0.001.

Both uncomplicated and complicated AD showed a statistically significant decrease in alpha diversity compared with controls (Figure 2).

### Principal component analysis

**FIGURE 3:**
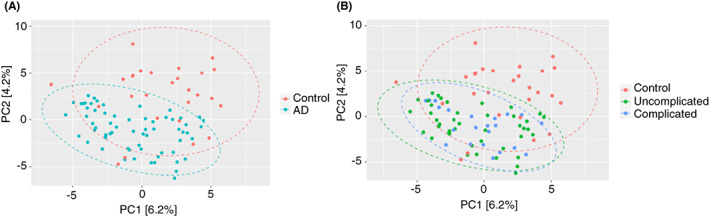
Principal component (PC) analysis using PC1 and PC2 showing separation between (A) acute diverticulitis (AD) and control groups and (B) complicated, uncomplicated, and control groups.

Principal components (PCs) 1 and 2 accounted for 6% and 4% of the total variation, respectively, with the remaining components contributing significantly less to the variation seen. These two PCs were used to generate Figure 3.

This analysis shows clear separation between all AD cases and controls (Figure 3A), but no separation between complicated and uncomplicated AD (Figure 3B). Permanova showed that our samples grouped significantly between disease states (AD versus control) but this grouping only explains 2.9% of the variance (adonis: *R* ^2^ = 0.029, *p* = 0.001). We also showed that there was a significant difference between within‐group dispersions (*p* = 0.001) possibly affecting PERMANOVA analysis, although our ordination supports the separation between AD and control group states.

### Microbiome results

**FIGURE 4:**
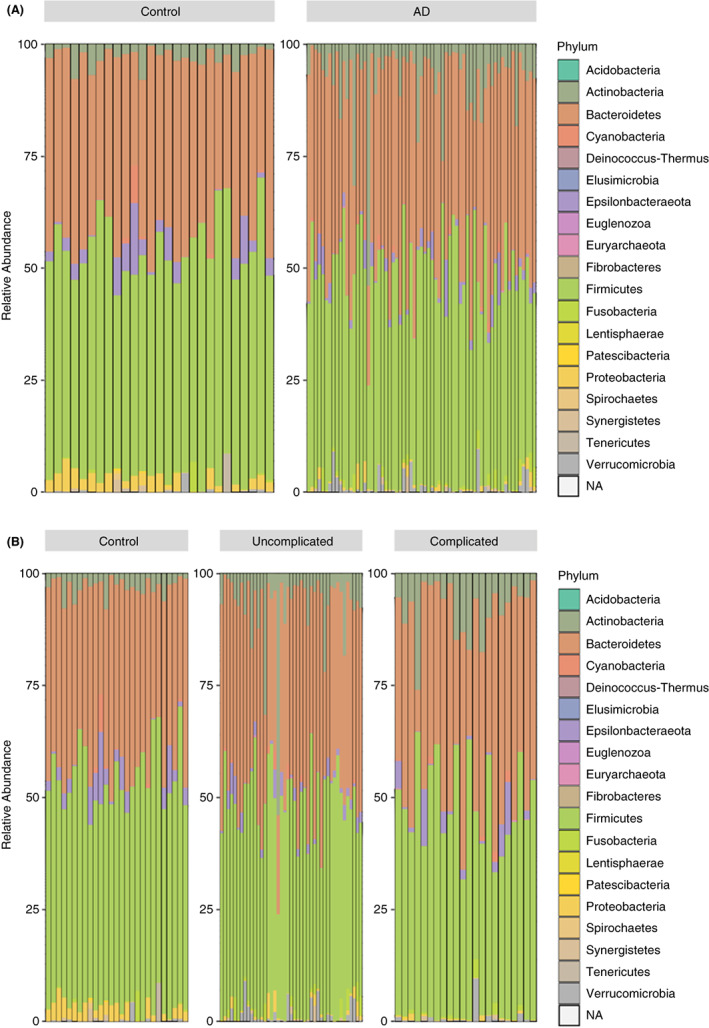
Relative abundance of phyla (A) between acute diverticulitis (AD) and controls and (B) between complicated AD, uncomplicated AD and controls, expressed as a percentage of the total taxonomic composition for each individual included in the study.

When comparing patients with AD (including subgroup comparisons of uncomplicated AD and complicated AD) with the control group using Wilcoxon tests there was a significant difference between the percentage abundances of the phyla Actinobacteria (AD versus control *p* < 0.001; complicated AD versus control *p‐*adjust = 0.001; uncomplicated AD versus control *p‐*adjust = 0.011) and Proteobacteria (AD versus control *p* < 0.0001; complicated AD versus control *p‐*adjust <0.001; uncomplicated AD versus control *p‐*adjust <0.001). Figure 4(A) shows the relative abundance of phyla between AD and controls and Figure 4(B) that between complicated AD, uncomplicated AD and controls.

### Differential abundance of genera

**FIGURE 5:**
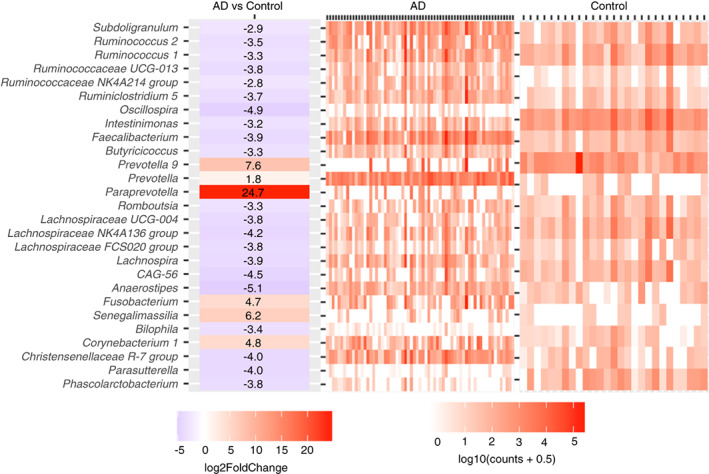
Log_2_ fold‐change of differentially abundant genera between acute diverticulitis (AD) and controls (left), and normalized abundances per sample in a group (right). Counts are DESeq2 normalized counts with an additional pseudocount of 0.5.

The heatmap in Figure 5 shows the differentially abundant genera between AD patients and controls. The heatmap on the left shows the log_2_ fold‐changes between AD and controls ((+) value = abundant in AD; (−) value = abundant in controls), while the heatmap on the right shows the abundance of these significant genera across samples with AD or from controls.

There were significant differences in microbiome composition noted for AD overall compared with controls. Twenty‐one genera were found to be significantly decreased in abundance in AD compared with controls, and were predominantly known commensal bacterial families and genera, such as Lachnospiraceae, *Ruminococcus* and *Faecalibacterium*, and contain species producing short‐chain fatty acids (SCFAs) [21, 22]. Only six genera were significantly more abundant in AD than in controls, and these included genera with known pathogenic roles, including *Fusobacteria*, *Prevotella* and *Paraprevotella* [23, 24, 25, 26]. Significance values for all differentially abundant taxa are included in Table S2.

**FIGURE 6:**
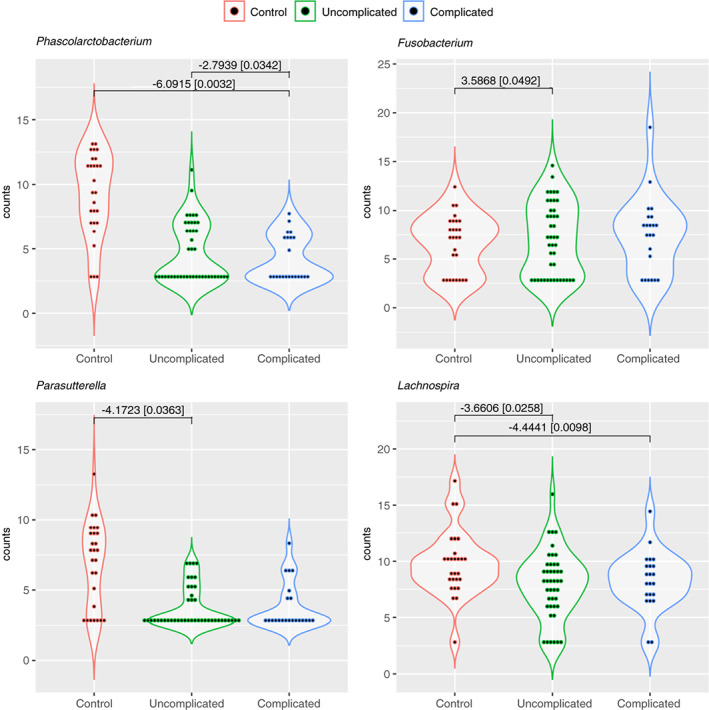
Violin plot demonstrating examples of differentially abundant genera comparing controls, uncomplicated acute diverticulitis (AD) and complicated AD with log2 fold‐change [*p*‐adjusted values].

Differences between uncomplicated AD and complicated AD were also observed, albeit less marked. In subgroup analysis, greater abundance of genera including *Prevotella*, *Fusicatenibacter* and *Faecalibacterium* were observed in the complicated group compared with uncomplicated AD (Figure 6).

## DISCUSSION AND CONCLUSIONS

This study demonstrates distinct compositional changes in the microbiome of AD, with decreased diversity and decreased abundance of commensal taxa in patients with AD compared with controls. In addition, several known pathogenic taxa were increased in abundance in AD compared with controls.

There is little published literature examining the microbiota of AD, with differing methodologies limiting direct comparison. Daniels et al. compared the microbiota from rectal swabs of 31 CT‐confirmed cases of uncomplicated AD with 27 controls [27]. In contrast to the current study, they observed increased alpha diversity in AD compared with controls, predominantly as a result of differences in the phylum Proteobacteria. This may be due to a confounding effect as a result of study design – while rectal swabs in the AD group were taken in the emergency department before administration of antibiotics, swabs in the control group were taken at the time of colonoscopy, implying use of MBP. The use of MBP has been shown to significantly affect the composition of the microbiota, notably reducing diversity [28, 29]. Furthermore, inclusion of patients with disease states including inflammatory bowel disease and benign and malignant neoplasms in the control group is again likely to bias results, as these states are associated with decreased diversity [30, 31].

Gueimonde et al. compared *Bifodobacterium* species from distal colonic mucosal biopsies from 21 individuals with colorectal cancer, nine with diverticulitis and four with inflammatory bowel disease [32]. There were distinct differences between the three groups, again indicating a role of the microbiota in gastrointestinal disease; however, the study was limited by its sample size and by only evaluating a single species of the microbiota. The colonic microbiota is complex and disease states are likely to result from whole microbiota dysbiosis. With the exception of a few solitary disease‐causing pathogens, studying single species can be misleading [33].

The microbiome of symptomatic uncomplicated diverticular disease (SUDD) has been examined. This entity, characterized by abdominal symptoms (pain, diarrhoea, bloating) in an individual with diverticulosis but without overt inflammation is associated in one study with decreased abundance of *Clostridium* cluster IX, *Fusobacterium* and Lactobacillaceae compared with controls [26]. In a second study, increased abundance of *Akkermansia muciniphila* was noted in both asymptomatic diverticulosis patients and SUDD compared with controls [34].

It is uncertain if the microbiome is altered in individuals with diverticulosis. Two studies have been published, both of which found no difference between those with diverticulosis and those without [35, 36]. These studies have limitations – both were single centre and both analysed the microbiome of individuals undergoing screening colonoscopy. This presents a possible source of bias through the affect of MBP on microbial makeup. There is also potential for selection bias in the study by Jones et al. [35] because participants were undergoing screening colonoscopy, which in the USA requires health insurance and therefore this population may not be representative of the general population.

Disease states related to microbiome changes arise through two mechanisms, firstly through the presence (or increased abundance) of disease‐causing microbiota and secondly through decreased abundance and reduced diversity of commensal microbiota [33]. In the current study, patients with AD had a decreased abundance of recognized commensal genera, including those that produce SCFAs (propionate, acetate and butyrate). SCFAs provide 70% of the energy requirement of colonocytes; abundance of these molecules supports the metabolic demands of the cell, enabling it to maintain the intestinal barrier through improved tight‐junction integrity and mucin production [37, 38]. The luminal concentration of SCFAs has been directly correlated with the thickness of the mucous layer in animal studies [39]. SCFAs also enhance cellular metabolism in lymphocytes, with proven increases in lymphocytes, supporting activation, plasma‐cell differentiation and antibody production [40]. Loss of commensal microbiota genera and their by‐products leads to impairment of the intestinal barrier, leading to increased microbiota–immune cell interactions. Combined with downregulation of anti‐inflammatory processes, a proinflammatory state can ensue.

There were several taxa with increased abundance in AD identified in the current study, including known pathogens. *Prevotella* is a large genus with over 40 species cultured, at least three of which are known to reside in the gut. These species are generally commensal, being especially predominant in individuals who consume diets high in plant‐based carbohydrates. However, these microbes are highly adaptable, responding to niches within the human body by modulating gene repertoires [41]. This fact, in combination with significant inter‐individual variability in microbe–host interactions, leads to differences in perceived interaction, from beneficial to detrimental. *Prevotella copri* has been shown to increase susceptibility to colitis in mice, and with increased cytokine production and chronic colonic inflammation in individuals with HIV [23, 24]. There was increased abundance of *Prevotella* in AD patients in the current study, and particularly in patients with complicated AD. Another genus with observed increased abundance was *Fusobacterium*, which is correlated with the pathogenesis of colorectal cancer through activation of host inflammatory responses [25, 42]. While the current study cannot provide evidence of causation, the authors propose the mechanism of pathogenic involvement as an alteration of the protective and pathogenic interactions between the microbiota, the colonic cells and the immune system. This alteration contributes, along with other established factors, to an individual’s propensity to develop diverticulitis. While these changes in the microbiota may exist throughout the colon, it is in the sigmoid colon where diverticula are predominantly found (in the West), where microbial density is higher and where stool transit time is slower, thus allowing for more microbiota–host interactions.

Evaluation of colonic flora has traditionally been obtained through faecal sampling; however, alternative means include mucosal biopsy and rectal swab. The optimum method is debated. There are differences in the microbiome present in the bowel lumen versus the mucous layer of the bowel wall. Some authors argue that only the mucosal microbiome is of clinical relevance, as most of the host–bacteria interactions occur at this level [43, 44, 45]. Analysis of the mucosal microbiome is more challenging as generally this is done with flexible endoscopy. This procedure is invasive, and usually requires MBP, which can in itself alter the composition of the microbiome [28, 29]. Rectal swab was used in the current study as it was minimally invasive and could be used at point of care, prior to the administration of antibiotics. Rectal swabs have been shown to correlate with faecal samples in previous studies [46, 47, 48, 49] and in a feasibility study undertaken by our group [50].

The strengths of the current study include the requirement for imaging confirmation of AD and the large sample size relative to published data. A limitation of the study is the single time‐point analysis, which provides only a snapshot in time. Observed changes in the microbiota may not reflect an individual’s long‐term microbiota, and may reflect a response to, rather than a contribution to, the pathogenesis of AD. However, a longitudinal study to resolve this would be impractical given the rarity of progression of diverticulosis to AD. We were unable to control for potential confounding due to comorbidity and for the presence of diverticulosis in the control group which could introduce bias. While this study is the largest in the field, the numbers are still relatively small, particularly for subgroup analysis, and from a single centre. A further limitation lies in 16S rRNA gene sequencing, which is currently limited to providing genus‐level information.

The pathogenesis of AD is complex with many protective and pathogenic factors identified. The current study has added to the evidence that the microbiota is another contributor, and has a similarly complex role. While this is a challenging area to study, an improved understanding of the potential role of the microbiota in the pathogenesis of AD has significant clinical importance and this subject warrants further investigation.

## AUTHOR CONTRIBUTIONS

Michael O’Grady: Study formulation, collection and processing of microbiome specimens, manuscript writing. Greg Turner: Study formulation, collection and processing of microbiome specimens, manuscript writing. Arielle Sulit: Statistical analysis. Frank Frizelle: Study formulation, supervision, manuscript writing. Rachel Purcell: Study formulation, supervision, manuscript writing.

## CONFLICT OF INTEREST

No conflict of interest declared for any author.

## FUNDING INFORMATION

Richard Stewart Scholarship 2020, Dunedin Basic Medical Sciences Trust.

## ETHICS STATEMENT

This study was approved by the University of Otago Human Ethics Committee (Health), reference H20/009 and registered with the Canterbury District Health Board Research Office, reference RO#20008. Written informed consent was obtained from patients.

## Supporting information


Table S1

Table S2

## Data Availability

The data that support the findings of this study are openly available in UOC_diverticulitis at https://gitlab.com/alsulit08/uoc_diverticulitis, reference number 32064025.
