# Supplementary material for: Distinct changes in the colonic microbiome associated with acute diverticulitis
Source: Colorectal Dis. 2022 Aug 11;24(12):1591–601. doi: 10.1111/codi.16271 (PMC10087140; doi:10.1111/codi.16271)
Supplement: Supplementary file 1 — Table S1 [file CODI-24-1591-s001.docx]

Supplementary Table S1

| **Supplementary Table S1. Parameters used for Filtering and Trimming in DADA2** | | |
| --- | --- | --- |
|  | **AD (+ diverticulosis)** | **Controls** |
| **truncLen (Fwd, Rev)** | 240, 200 | 0 (no truncation) |
| **maxEE** | 2 | 1 |
| **maxN** | 0 | 0 |
| **rm.phix** | True | True |
| **truncQ** | 2 | 2 |
