# Supplementary material for: Distinct changes in the colonic microbiome associated with acute diverticulitis
Source: Colorectal Dis. 2022 Aug 11;24(12):1591–601. doi: 10.1111/codi.16271 (PMC10087140; doi:10.1111/codi.16271)
Supplement: Supplementary file 2 — Table S2 [file CODI-24-1591-s002.docx]

**Supplementary Table S2. Differentially abundant microbes between Control, Uncomplicated AD, and Complicated AD groups showing abundance fold changes and corresponding p-adjusted values between the groups. A positive fold change refers to abundance in group2 over group 1.**

|  | | | | |
| --- | --- | --- | --- | --- |
|  | **Comparison** | | **Differential Abundance** | |
| **Genus** | **Group1** | **Group2** | **log2FoldChange** | **padj** |
| ***Agathobacter*** | Control | Uncomplicated | -3.41 | 0.0363 |
| ***Anaerococcus*** | Uncomplicated | Complicated | 1.12 | 0.0408 |
| ***Anaerostipes*** | Control | Complicated | -4.17 | 0.0019 |
| ***Anaerostipes*** | Control | Uncomplicated | -6.03 | 2.14E-08 |
| ***Anaerostipes*** | Uncomplicated | Complicated | 1.86 | 0.0247 |
| ***Bacteroides*** | Control | Uncomplicated | -3.14 | 0.0178 |
| ***Bacteroides*** | Uncomplicated | Complicated | 1.99 | 0.0233 |
| ***Bifidobacterium*** | Control | Uncomplicated | -3.22 | 0.0357 |
| ***Bifidobacterium*** | Uncomplicated | Complicated | 2.8 | 0.0044 |
| ***Bilophila*** | Control | Uncomplicated | -3.64 | 0.0258 |
| ***Blautia*** | Control | Uncomplicated | -2.9 | 0.0363 |
| ***Butyricicoccus*** | Control | Uncomplicated | -6.38 | 2.29E-08 |
| ***Butyricicoccus*** | Uncomplicated | Complicated | 3.74 | 8.01E-07 |
| ***CAG-56*** | Control | Complicated | -4.77 | 0.0104 |
| ***CAG-56*** | Control | Uncomplicated | -4.43 | 0.0165 |
| ***Christensenellaceae R-7 group*** | Control | Complicated | -4.17 | 0.0098 |
| ***Christensenellaceae R-7 group*** | Control | Uncomplicated | -3.85 | 0.0155 |
| ***Corynebacterium 1*** | Control | Complicated | 5.19 | 0.0032 |
| ***Corynebacterium 1*** | Control | Uncomplicated | 3.8 | 0.0203 |
| ***Dialister*** | Uncomplicated | Complicated | -1.64 | 0.0499 |
| ***Dorea*** | Uncomplicated | Complicated | 2.96 | 0.0003 |
| ***Erysipelatoclostridium*** | Uncomplicated | Complicated | 2.92 | 0.0293 |
| ***Erysipelotrichaceae UCG-003*** | Uncomplicated | Complicated | 2.5 | 0.0499 |
| ***Faecalibacterium*** | Control | Uncomplicated | -5.52 | 1.10E-06 |
| ***Faecalibacterium*** | Uncomplicated | Complicated | 2.86 | 0.0002 |
| ***Fusicatenibacter*** | Control | Uncomplicated | -3.37 | 0.0203 |
| ***Fusicatenibacter*** | Uncomplicated | Complicated | 3.24 | 0.0002 |
| ***Fusobacterium*** | Control | Uncomplicated | 3.59 | 0.0492 |
| ***Intestinimonas*** | Control | Uncomplicated | -3.36 | 0.0221 |
| ***Lachnospira*** | Control | Complicated | -4.44 | 0.0098 |
| ***Lachnospira*** | Control | Uncomplicated | -3.66 | 0.0258 |
| ***Lachnospiraceae FCS020 group*** | Control | Uncomplicated | -5.59 | 0.0003 |
| ***Lachnospiraceae FCS020 group*** | Uncomplicated | Complicated | 3.03 | 0.0034 |
| ***Lachnospiraceae NK4A136 group*** | Control | Complicated | -4.47 | 0.0098 |
| ***Lachnospiraceae NK4A136 group*** | Control | Uncomplicated | -4.05 | 0.0165 |
| ***Lachnospiraceae UCG-004*** | Control | Complicated | -3.86 | 0.0494 |
| ***Lachnospiraceae UCG-004*** | Control | Uncomplicated | -3.83 | 0.0258 |
| ***Oscillospira*** | Control | Uncomplicated | -5.21 | 0.0197 |
| ***Parabacteroides*** | Uncomplicated | Complicated | 1.98 | 0.0499 |
| ***Paraprevotella*** | Control | Complicated | 23.74 | 7.90E-16 |
| ***Paraprevotella*** | Control | Uncomplicated | 24.83 | 2.17E-19 |
| ***Parasutterella*** | Control | Uncomplicated | -4.17 | 0.0363 |
| ***Phascolarctobacterium*** | Control | Complicated | -6.09 | 0.0032 |
| ***Phascolarctobacterium*** | Uncomplicated | Complicated | -2.79 | 0.0342 |
| ***Prevotella*** | Control | Complicated | 2.51 | 0.0054 |
| ***Prevotella*** | Uncomplicated | Complicated | 1.47 | 0.008 |
| ***Prevotella 9*** | Control | Uncomplicated | 8.09 | 0.0203 |
| ***Romboutsia*** | Control | Uncomplicated | -4.14 | 0.013 |
| ***Ruminiclostridium 5*** | Control | Uncomplicated | -3.82 | 0.0069 |
| ***Ruminiclostridium 5*** | Uncomplicated | Complicated | 4.87 | 2.36E-10 |
| ***Ruminococcaceae NK4A214 group*** | Control | Uncomplicated | -2.98 | 0.0258 |
| ***Ruminococcaceae UCG-002*** | Uncomplicated | Complicated | 1.82 | 0.0499 |
| ***Ruminococcaceae UCG-013*** | Control | Complicated | -4.83 | 0.0042 |
| ***Ruminococcaceae UCG-013*** | Control | Uncomplicated | -3.41 | 0.0317 |
| ***Ruminococcus 2*** | Control | Complicated | -5.43 | 0.0042 |
| ***Ruminococcus 2*** | Uncomplicated | Complicated | -2.35 | 0.0499 |
| ***Subdoligranulum*** | Control | Uncomplicated | -3.81 | 0.0069 |
| ***Subdoligranulum*** | Uncomplicated | Complicated | 1.8 | 0.0499 |
